# Disembodied Language in Early-Onset Schizophrenia

**DOI:** 10.3389/fpsyt.2022.888844

**Published:** 2022-07-05

**Authors:** Przemysław Zakowicz, Maria Skibińska, Joanna Pawlak

**Affiliations:** ^1^Department of Psychiatric Genetics, Poznan University of Medical Sciences, Poznan, Poland; ^2^Center for Child and Adolescent Treatment in Zabór, Zielona Góra, Poland

**Keywords:** psychosis, disembodiment, language, phenomenology, schizophrenia

## Abstract

A recent view on schizophrenia phenomenology underlines the impaired relations between the mind and the body. An aberrant feeling of ipseity may be the real source of suffering of the patients from psychosis and impacts general symptomatology. The disturbed connection between thinking processes and environmental stimuli may lead to language disembodiment. In the study, we aimed to experimentally test the presence of disembodied language and investigate its association with symptoms of psychosis in adolescents diagnosed with early-onset schizophrenia spectrum disorders. Assessment of language embodiment was conducted using the Zabór Verbal Task (ZVT) with concurrent linguistic and clinical assessment using the Thought, Language, and Communication Scale (TLCS) and Positive and Negative Symptoms Scale (PANSS). The study group of patients (*n* = 31) aged 11–18 years, with the diagnosis of schizophrenia spectrum according to Diagnostic and Statistical Manual of Mental Disorders, fourth edition (DSM-IV) and the International Classification of Diseases (ICD-10) criteria, was compared with a sex- and age-matched healthy control sample (*n* = 31). Patients with psychosis made more errors in ZVT than healthy controls (*p* = 0.01) and this parameter did not improve after 6–8 weeks of standard treatment (*p* = 0.55). A higher number of errors in ZVT were associated with the presence of auditory hallucinations (odds ratio [OR] 1.14; 95% CI 1.02–1.26). ZVT errors coincided with perception disorders, alternatively to the TLCS scores where we observed association with abnormal beliefs. The results of these preliminary studies indicate the value of the phenomenological approach in the diagnosis of schizophrenia spectrum and suggest a potential involvement of language disembodiment in symptomatology.

## Introduction

Schizophrenia is characterized by experiencing false perceptions and beliefs (1). Current phenomenological literature emphasizes the concept of schizophrenia as the self (ipseity) disturbance (1). The term *ipseity* is understood as the minimal, subjective feeling of self and decides about the perception of reality as one's own experience (2). Ipseity Disturbance Model (IDM) proposed by Sass and Parnas (3) explains schizophrenic symptoms regarding three phenomena: hyper-reflexivity (forced self-orientation leading to aberrant salience), diminished self-presence is defined as the loss of subjectivity in the feeling of awareness, and disturbed grip or hold leads to perceptual aberrancies (3, 4). IDM finds reflection in clinical data. The use of Examination of Anomalous Self-Experience (EASE), the standardized questionnaire for patients with a schizophrenia diagnosis, provides a clear dissection of schizophrenia symptomatology from the bipolar psychotic state (4). Significant disturbance of ipseity may also be present in other schizophrenia spectrum disorders and at-risk mental state (5).

Ipseity disturbance has been therefore suggested as the basic psychotic state, which leads to secondary phenomena, such as positive symptoms and loss of body awareness (6). The subjective feelings of the body change during schizophrenia (disembodiment), leading to misattribution of inner states, normal feelings, uncoupling with bodily representations, which may lead to extraordinary, delusional explanations (7).

Stanghellini, in the literature review (2009) (8), emphasizes disembodiment as the core feature of schizophrenia and subdivides it into three domains: disembodied of the self, disembodiment of self-object relations, and disembodiment of intersubjectivity. In the first domain, the patient due to disembodied view of the organism reveals the conviction about the deaminated body (8). The primary problem of disrupted self-awareness impacts daily activities and the relation to the treatment schedule (9). Lack of integrated representation of self also generates further metacognitions, such as poorer levels of self-reflectivity (10). The second domain described by Stanghellini fasters changed the meaning of usual objects for the patients with schizophrenia, who loses clear representation of the external world. The third one, intersubjectivity, refers to the combination of somato-psychic and auto-psychic depersonalization, leading to social isolation, observed as schizophrenic autism (8). Symptoms of schizophrenia may be a direct consequence of disembodiment due to problems in the recognition of familiar patterns in perception (11).

Perceptual and thinking aberrancies may have roots in disembodiment affecting the structure of a patient's language (disembodied language). As reported in previous studies, linguistic abnormalities are a core feature of schizophrenia (12), may serve as a marker, especially in the early stages of the disease (13), and predict conversion from at-risk mental state to schizophrenia. The potential role of language disembodiment in psychotic phenomenology derives from linking the language disturbances with the concept of ipseity.

The concept of language embodiment is based on a bi-directional hypothesis and connects somatic representations of sensory or motor perceptions with language semantics (14, 15). This mechanism activates bodily experiences in the inner, solipsistic simulation of the reality with the use of linguistics (Perceptual Symbol System, PSS) (16). PSS explains the role of language information in priming the motoric response, such as in the study by Zwaan et al. (17), where participants presented with a sentence (containing spatial modalities) tend subsequently to respond faster to matching visual stimuli. Hence, the hypothesis about the occurrence of language-induced simulation precedes and enables proper reaction. Similar results were obtained after the presentation with the shape and motion of an object (18).

There is a lack of studies on language embodiment assessment in schizophrenia. The available approach explored mind-body connection in the communicational aspect in patients with schizophrenia, who tend to exhibit difficulties in understanding emotional body language; deficits corresponded with the severity of the disease's symptoms (19). Inner and subjective processing of language not only appears as a possible aspect of general linguistic disturbances in psychosis but may also lead to particular secondary phenomena, such as auditory verbal hallucinations (AVHs; misattribution of inner speech) (20). From this point of view, the assessment of disembodied language seems to be an essential paradigm in further understanding schizophrenia psychopathology.

### Aim of the Study

In the study, we searched the evidence for the presence of language disembodiment in patients diagnosed with schizophrenia-spectrum disorders and tracked the relationship between potential deficits in language embodiment and general symptomatology. To decrease the impact of chronic disorder and treatment on cognitive and communication processes, we investigated a group of adolescents (11–18 years old). Moreover, the onset of schizophrenia before 18 years of age (early-onset schizophrenia) makes the investigated group more homogenous and with stronger biological root of psychosis (21). We assumed the primary hypothesis about (i) the presence of deficits in embodied language among patients with psychosis vs. healthy controls and secondary, (ii) association of language disembodiment with psychotic symptoms (particularly for AVHs and thought insertion phenomena) and linguistic deficits assessed with Thought, Language, and Communication Scale (TLCS). To our knowledge, this is the first study to assess the youth population of patients with psychosis in this context.

## Materials and Methods

### Study Design

The study was designed and conducted by the appointment of the local bioethics commission at Poznań University of Medical Sciences (1,066/19). Inclusion in the study was preceded by informed consent of the adolescent and/or a caregiver. All consecutive patients with an initial diagnosis of schizophrenia were admitted to the Center for Child and Adolescent Treatment, Zabór (tertiary referral children and adolescent psychiatric center for western Poland) were proposed to participate in the study. The healthy control group was recruited from the local community, in cooperation with primary care units. Inclusion criteria for the study group were as follows: psychotic episode meeting diagnostic criteria for schizophrenia (*n* = 25), schizophreniform (*n* = 1) disorder, or non-otherwise specified psychotic disorder (*n* = 5). The diagnosis was made by consensus of two child and adolescent psychiatrists and evaluated with the use of Structuralized Clinical Interview for Diagnostic and Statistical Manual of Mental Disorders, fourth edition (DSM-IV) (SCID-I) (22). Lifetime symptomatology was assessed using Operational Criteria for Psychotic Symptoms (OPCRIT) (23). Exclusion criteria for the study group were established to disclose organic or secondary psychosis with similar symptoms and medical states that might impact cognitive functioning and were as follow: abnormalities in the basic laboratory blood tests (full blood count, thyroid hormones(thyroid function tests [TSH], free T3 [fT3], and free t4 [fT4]) (24) and inflammatory markers[C-reactive protein [CRP], sedimentation rate)], acute intoxication or withdrawal state regarding alcohol or psychoactive substances (verified with the urinary test) (25), and medical states requiring immediate intervention [exacerbation of a chronic disease, post-operational state, (26)]. For the healthy control group, exclusion criteria were as follows: past or current history of psychiatric disease (verified with Mini International Neuropsychiatric Interview, MINI 7.0.2) (27), familiar burden with psychiatric disease in first- and second-degree relatives, medical states requiring immediate intervention (as above), abnormalities in the basic laboratory blood tests (as listed above), and acute intoxication or withdrawal state from psychoactive substances. The clinical assessment of patients took place at two time points: up to 7 days after admission to the hospital and after 6–8 weeks of hospitalization. The sample was chosen based on the inclusion of all patients with psychosis admitted to the tertiary center within the recruitment period. The healthy control group was evaluated once. All participants were unrelated.

### Instruments

Thought, Language, and Communication Scale, Zabór Verbal Task (ZVT), and Positive and Negative Symptoms Scale (PANSS) were applied at both time points to assess linguistic and clinical symptoms.

Thought, Language, and Communication Scale was introduced by Nancy Andreasen in 1979 and serves as a reliable assessment of formal thought disorder by the clinician (28). The scale consists of twenty items: poverty of speech, poverty of speech content, the pressure of speech, distractible speech, tangentially, derailment, incoherence, illogicality, clanging, neologisms, word approximations, circumstantiality, loss of goal, perseveration, echolalia, blocking, stilted speech, self-reference, and paraphasia (phonemic and semantic). Each item is assessed numerically from absent “0” to extremely severe “3–4”(28), 0–4 for 1–9 and 20th item, and 0–3 for 10–19th item. In the study, we assessed the speech of the patient in the first time point (standard psychiatric examination and anamnesis, about 30 min of conversation) and in the second time point (psychiatric examination and summary of the hospitalization, similarly).

Zabór Verbal Task is the author's own, non-published so far clinical tool designed to assess language embodiment based on the spatial value of words. The task consists of the board (60 cm × 30 cm) placed in the central area of the patient's visual field. The board contains seven rectangle boxes in three groups, respectively, to the number of word cards. The patient after receiving a command, *please place the words on the board as you think they should be*, sticks each word to one of the boxes (the procedure was described in the Supplementary Material).

Positive and Negative Symptoms Scale is an operationalized rating questionnaire to assess the presence and severity of positive, negative, and general symptoms in schizophrenia, validated clinically and used clinically since the 80 s (29).

### Statistical Analysis

The normality of the data was checked by plotting histograms for all variables and graphical appreciation of distributions. None of the variables tested followed normal distribution, which was further confirmed using the Kolmogorov-Smirnoff test. The Mann-Whitney U test was used to compare ZVT and TLCS results in psychosis vs. control group and the presence of specific symptoms based on SCID and OPCRIT items. The Wilcoxon signed-rank test was used to compare ZVT, TLCS, and PANSS scores in patients with psychosis before and after the treatment. The relationship between AVH and thought insertion phenomena and ZVT scores was analyzed using logistic regression. Spearman's correlation was applied in the analyses of correlation rates between psychometric numeric scales. Internal consistency of ZVT was tested using Cronbach's alpha. Analyses were conducted using the Statistica v13 software. Statistical significance was set at 0.05. The sample size was calculated based on the allowed margin of error of 5% and the proportion of the psychotic population was set at 3% (lifetime prevalence of psychosis) (30), with *N* = 45 as a result, ± 15% was allowed for dropouts and missing data.

## Results

### Descriptive Characteristics of the Study Group

Detailed demographic and clinical data of the studied populations are presented in Table 1. Most of the included subjects met the diagnostic criteria for paranoid schizophrenia (*n* = 25, 80.65%). Near half of the patients (*n* = 13) presented a single episode with partial remission, ten patients were identified to have prominent residual symptoms. The patients were administered with neuroleptic treatment with the most frequent aripiprazole (*n* = 17, 54.83%) and mean chlorpromazine was equivalent to 330.34 mg ( ± 154.68 mg), 4 patients dropped out before 6 weeks of treatment due to discharge on caregiver's demand. A past history of cannabinoid misuse was reported by 3 patients and harmful alcohol consumption by 2.

**Table 1:** Demographic and clinical data (SCID items).

**Early onset psychosis (*****n*** **=** **31)**		**Healthy control (*n =* 31)**
Age (min.-max; mean^*^; ±SD)	11–18; 14 (±1.94)	12-18; 15 (±1.67)
Sex^**^	Male (*n =* 15), female (*n =* 16)	Male (*n =* 13), female (*n =* 18)
Family history of schizophrenia	4	*ex*.
Family history psychiatric disease in I and II degree	14	*ex*.
Disease type (*n*; %)	SCH (*n =* 25; 80.65), SF (1; 3.23), NOS (5; 16.13)	
**Descriptive psychopathology: Number of patients with a symptom (%)**
**Delusions**
Reference	19 (61.29)
Persecutory	14 (45.16)
Grandiose	1 (3.23)
Somatic	2 (6.45)
Religious	5 (16.13)
Being controlled	22 (70.97)
Bizarre	19 (61.29)
**Hallucinations**
Auditory	19 (61.29)
Visual	14 (45.16)
Tactile	7 (22.58)
Other	4 (12.90)
**Negative symptoms**
Avolition	27 (87.10)
Alogia	26 (83.87)
Affective blunting	30 (96.77)

*SCH, schizophrenia; SF, schizophreniform disorder; NOS, psychotic disorder none otherwise specified; ex, exclusion criterion*.

* *p = 0.13*,

** *p= 0.38*.

### Linguistic Parameters in Relation to Psychotic Symptoms

#### Psychometric Assessment

Mean PANSS total values were reached 106.00 (± 16.51) at the admission (T_0_) and 55.52 (± 15.44) in follow-up (T_1_; *p* < 0.01). Regarding the parameters of language assessment, mean TLCS values were reached 18.87 (± 10.67) in T_0_ and 5.86 (± 6.16) after treatment (*p* < 0.01), the TLCS score differed significantly between patients with schizophrenia and the healthy control group (*p* < 0.01). The TLCS score in T_0_ was moderately correlated with PANSS positive symptoms (*R* = 0.36, *p* = 0.03), for TLCS in follow-up, strong correlation was observed with all PANSS sub-dimensions (positive: *R* = 0.53, *p* < 0.01, negative: *R* = 0.63, *p* < 0.01, general: *R* = 0.54, *p* < 0.01) and PANSS total score (*R* = 0.58, *p* < 0.01). ZVT score for both admission and follow-up assessment was occurred not to correlate at a significant level with positive and negative symptom scores. In the relation between TLCS and ZVT, moderate negative correlation was observed between TLCS T_0_ and ZVT T_0_ (*R* = −0.36, *p* = 0.04).

#### ZVT and Psychotic Symptoms: Thought Insertion Phenomena and AVH

**Figure 1:**
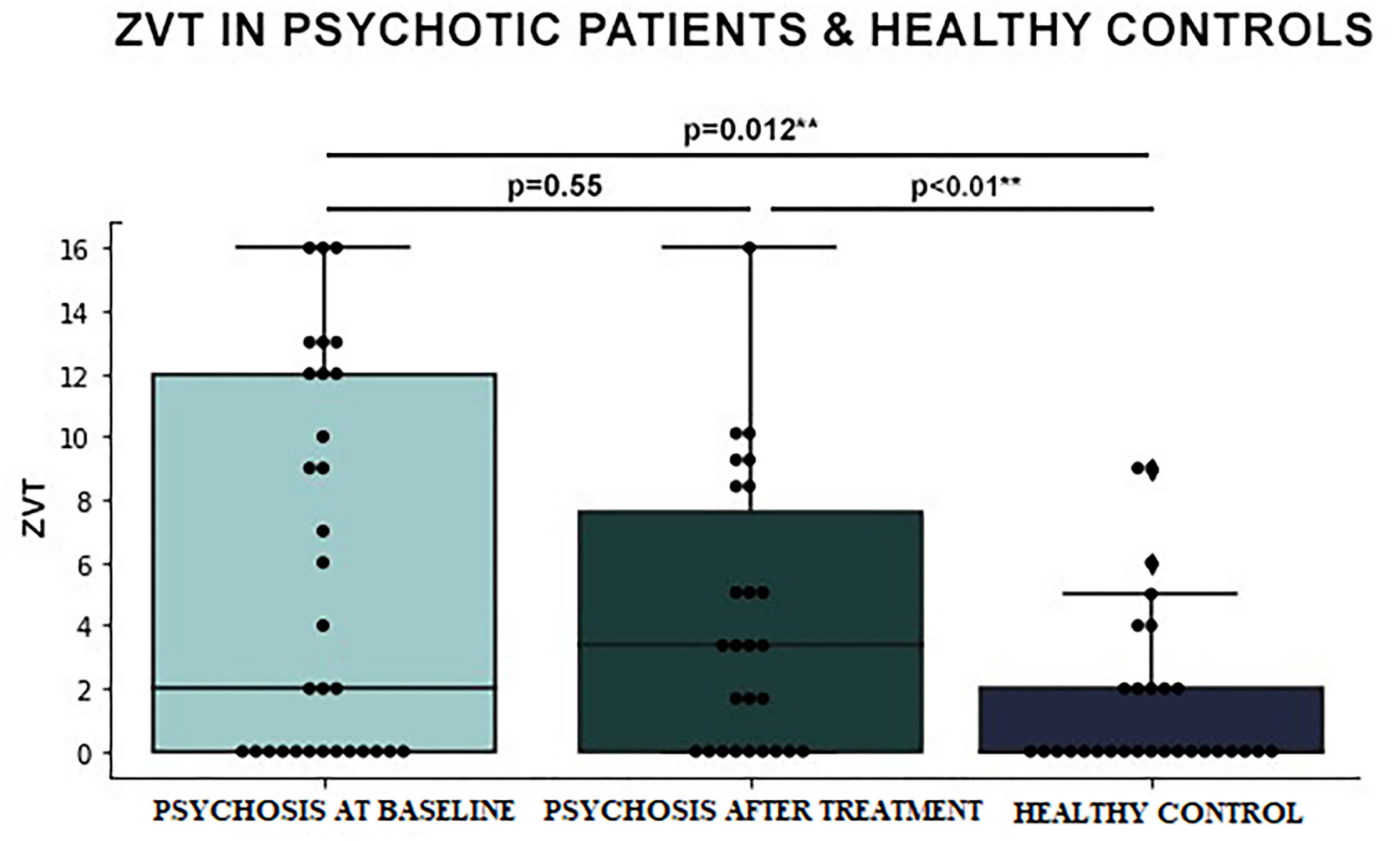
Differences in Zabór Verbal Task (ZVT) results between early-onset psychosis patients at baseline vs. after treatment vs. healthy controls (logistic regression).

Internal consistency for the ZVT was assessed using Cronbach alpha, revealing high consistency (α = 0.93). The mean of ZVT was 2 in the psychotic vs. 0 in the healthy control group (*p* = 0.01). There was major skewness toward the 0 value. It was an expected finding given the fact that most healthy children were expected to solve the task without mistakes. The ZVT results did not significantly differ before and after antipsychotic treatment in the patient group (*p* = 0.55; Figure 1).

In logistic regression modeling, we search for the link between ZVT scores, AVH, and thought insertion phenomena (based on SCID data). For auditory hallucinations, the model was significant at *p* = 0.016 and could explain 12.7% of the variance (Nagelkerke R^2^) and correctly classified 73.8% of cases (odds ratio [OR] 1.14; 95% CI 1.02–1.26). For thought insertion, no significant model was obtained.

#### Differences in Relation to TLCS and ZVT Results in Symptoms

In the Mann-Whitney U test, abnormalities in ZVT occurred to coincide mainly with *abnormal perception* in OPCRIT, TLCS score was associated with the *thought content* module. ZVT errors in both T_0_ and T_1_ were observed in patients experiencing persecutory voices (Z = 2.0, *p* < 0.01 for T_0_ and Z = 2.01, *p* = 0.04 for T_1_) and third person auditory hallucinations (Z = 1.97, *p* = 0.05 for T_0_ and Z = 2.18, *p* = 0.03 for T_1_). Higher TLCS T_0_ scores were observed in patients with bizarre delusions (Z = 2.13, *p* = 0.03), thought insertion delusions (Z = 1.96, *p* = 0.05), thought withdrawal (Z = 2.14, *p* = 0.03), and thought broadcasting delusions (Z = 2.17, *p* = 0.03). Conversely, a negative coincidence of TLCS T_0_ scores was observed in patients experiencing: other primary delusions (Z = −3.37, *p* < 0.01), primary delusional perception (Z = −2.46, *p* = 0.01), and persecutory voices (Z = −1.99, *p* = 0.05). Detailed data are presented in Table 2.

**Table 2:** Mann-Whitney U analysis of Zabór Verbal Task (ZVT) and Thought, Language, and Communication Scale (TLCS) scores in relation to psychotic symptoms (Operational Criteria for Psychotic Symptoms [OPCRIT] items).

	**ZVT_T0**		**ZVT_T1**		**TLCS_T0**		**TLCS_T1**	
	**Z**	**p**	**Z**	**p**	**Z**	**p**	**Z**	**p**
**Abnormal perceptions**
Thought echo	1.29	0.19	1.27	0.2	0.88	0.38	0.95	0.34
Third person auditory hallucinations	2.04	**0.04**	2.23	**0.03**	0.02	0.99	0.07	0.94
Running commentary voices	1.8	0.07	1.5	0.13	0.75	0.45	0.07	0.94
Abusive/accusatory/persecutory voices	3.0	**<0.01**	2.05	**0.04**	−1.99	**0.05**	0.33	0.74
Other (non–affective) auditory hallucinations	1.03	0.3	0.05	0.96	1.62	0.11	−1.37	0.17
Non–affective hallucination in any modality	0.63	0.53	0.1	0.92	1.77	0.08	−0.51	0.61
**Abnormal thought content**
Persecutory delusions	−0.28	0.78	−0.78	0.44	−1.07	0.28	−0.68	0.50
Delusions of influence	0.12	0.91	−0.17	0.86	0.67	0.50	−1.22	0.21
Bizarre delusions	−0.35	0.74	0.58	0.56	2.13	**0.03**	−0.71	0.48
Delusions of passivity	0.61	0.22	0.68	0.50	0.45	0.65	−1.21	0.22
Primary delusional perception	0.18	0.86	−0.74	0.46	−2.46	**0.01**	1.39	0.17
Other primary delusions	−0.26	0.79	0.59	0.55	−3.38	**<0.01**	0.21	0.83
Thought insertion delusions	−0.39	0.70	−0.85	0.39	1.97	**0.05**	−0.77	0.44
Thought withdrawal delusions	0.04	0.97	−0.40	0.70	2.14	**0.03**	−0.17	0.86
Thought broadcasting delusions	0.02	0.98	−0.56	0.57	2.17	**0.03**	0.02	0.99

*Bolded: statistically significant*.

## Discussion

The study presents the preliminary analysis of disembodied language assessment in relation to psychotic symptoms, based on widely used clinical tools. To assess language disembodiment, we used authors' test (ZVT). We aimed to construct an easy-applicable clinical tool to assess language disembodiment. In the manuscript, we focused on the link between disturbed spatial understanding of language and clinical characteristics of early-onset schizophrenia spectrum disorders.

Early-onset psychosis population differed significantly from the healthy control group in the outcomes of ZVT and TLCS. The performance of ZVT did not correspond with general improvement after standard pharmacotherapy and was associated with the presence of auditory hallucinations. In relation to experienced symptoms, ZVT occurred to be connected with aberrant perception, whereas TLCS deficits corresponded with delusional thought content.

The language of patients with schizophrenia has been studied so far that thoroughly include semantic and syntax aspect, such as automatic language analysis (31). Recent research indicates the link between language disturbance and negative symptoms (32). In our study, obtained data suggest coincidence regarding hallucinations and delusional ideation. Beyond reliable data on linguistics, using phenomenology-derived concepts, such as embodiment, may improve schizophrenia diagnosis in an early age. What has been shown in our study, disembodiment appears to be a parallel dimension of disturbed language, linked with auditory hallucinations.

Here, we understood the language disembodiment as a part of general disembodiment due to self-disturbances (SD) (3). Recent data suggest SD as a trait phenomenon, remaining a constant domain of psychopathology (2) and a potential marker of schizophrenia spectrum (33). Empirical studies using EASE revealed a significant coincidence of self-disorder with typical formal thought aberrancies and imagination anomalies (34), which suggests the crucial role of the self in language processing. An embodied approach to language defines concepts as having the same structure as perceptions, such as the color, shape, movement, and emotional value (35). In the study, we have used spatial properties.

Observed differences in word placing may also be explained alternatively than as the part of the disordered self. A major confounding factor is the use of word semantics during the task. Presented deficits may not reflect spatial (somatic) representation of words, but aberrant semantic networks are evidenced for patients with psychosis (36) and semantic priming effect (37). This effect enables faster decision-making due to presentation with context information, hence in our study, preceding words could induce choosing the other one as the next in the procedure of placing. Word placing schedule may be also affected by deficits in working memory and decision-making typically present in schizophrenia. As it was shown in word-fluency tests, deficits in working memory affect the outcome of language abilities (38).

Zabór Verbal Task outcome is shown to be connected with the presence of visual and auditory hallucinations, initial thought, language and communication deficits, and initial rate of positive symptoms. The performance of ZVT did not correspond with general improvement after standard pharmacotherapy. From the results we obtained, embodied language might potentially serve as a neuropsychological biomarker of psychosis, independent from the state of exacerbation/remission, but present and characteristic of the disease. This direction requires further studies.

**Figure 2:**
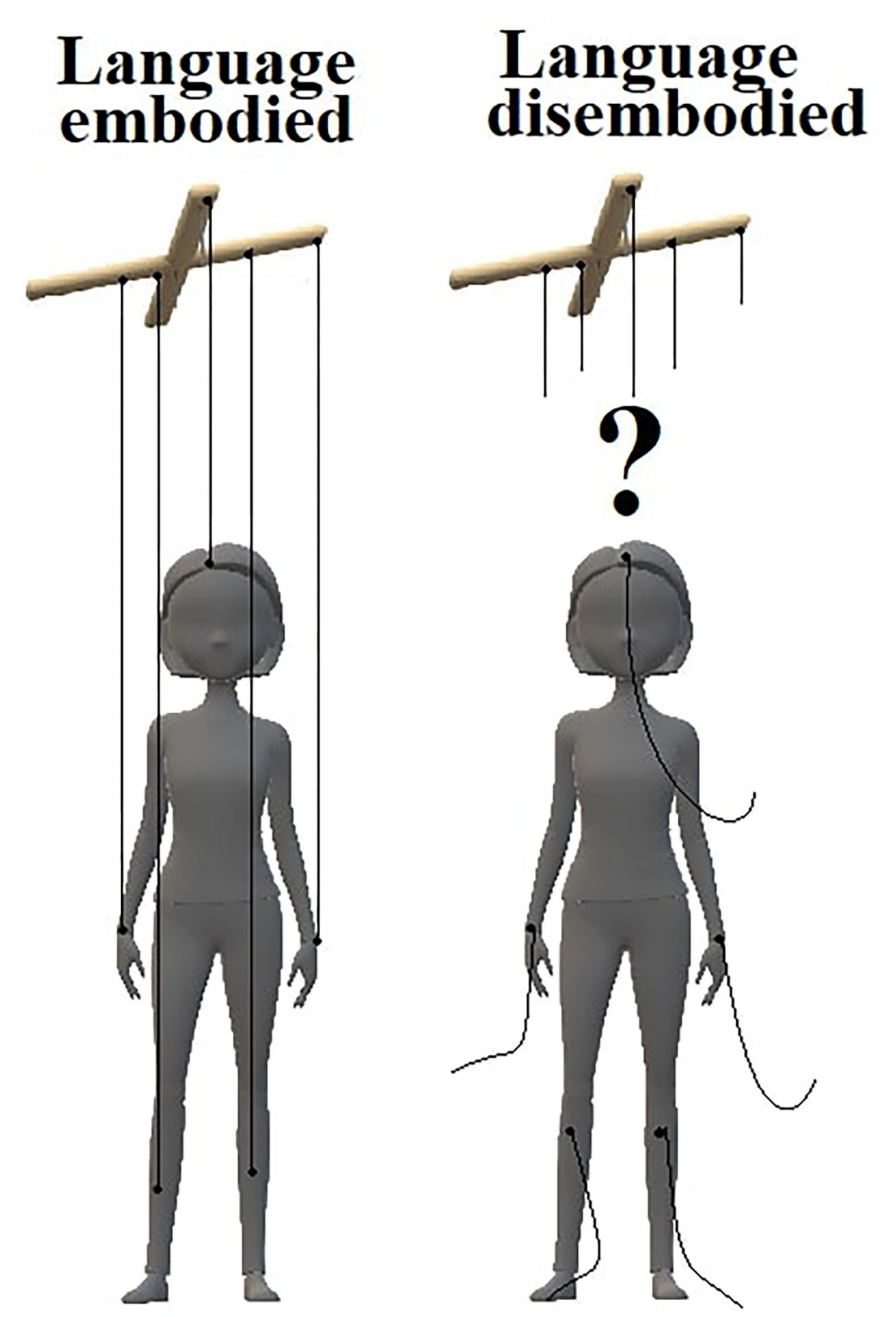
An impaired embodiment of language may lead to compensatory delusional belief about external influence on patient's inner processes. In a healthy state **(left)**, the connection of language with bodily sensations provides self-integrity. Patients with schizophrenia **(right)**, due to disrupted language-body connection, may perceive their own thoughts as unfamiliar.

Results of this preliminary study indicate disembodied language as a clinically valuable parameter in the general psychopathology of schizophrenia. Further studies need to find an answer about the relation of language disembodiment with other thought disorders. The open question is the primacy of disembodiment before psychotic symptoms or secondary character of disembodiment. Following this approach, typical for schizophrenic patients' delusionary feelings of external influence, such as thought insertion or thought withdrawal, may be the direct consequence of disturbed ipseity (2) and language disembodiment (see Figure 2). Such an approach underlines the role of subjective experience in treatment. Current perception of remission in schizophrenia regards more often explicit and visible positive symptoms and does not provide better self-integrity of patients (6); as has been shown in our study, standard neuroleptic treatment did not show benefits in the spatial understanding of language, hence future therapeutic interventions for people at risk to develop schizophrenia may benefit from self-directed psychotherapy (39).

The study is not free from limitations. We did not assess the TLCS objectively, by recording the patient's utterance before analysis due to ethical concerns. Instead of this, the utterance was assessed directly after the examination, which is prone to subjectivity. The second limitation is the number of participants included. Several scales for clinical description (such as EASE) were omitted in the investigation of ZVT correlation with other symptomatology. Hence the preliminary character of the outcomes is the purpose for further studies the wider population.

## Data Availability Statement

The original contributions presented in the study are included in the article/Supplementary Material, further inquiries can be directed to the corresponding author.

## Ethics Statement

The study was designed and conducted by the appointment of the Local Bioethics Commission at Poznan University of Medical Sciences (1066/19). Written informed consent to participate in this study was provided by the participants' legal guardian/next of kin.

## Author Contributions

PZ, MS, and JP: methodology and manuscript preparation. PZ: recruitment and clinical evaluation. MS: computations. All authors contributed to the article and approved the submitted version.

## Funding

The research was financed from the large research grant from statutory funding for young researchers–doctoral students for 2021 and Department of Psychiatric Genetics statute sources: 502-20-22196440.

## Conflict of Interest

The authors declare that the research was conducted in the absence of any commercial or financial relationships that could be construed as a potential conflict of interest.

## Publisher's Note

All claims expressed in this article are solely those of the authors and do not necessarily represent those of their affiliated organizations, or those of the publisher, the editors and the reviewers. Any product that may be evaluated in this article, or claim that may be made by its manufacturer, is not guaranteed or endorsed by the publisher.

## References

[1] Rasmussen AR Raballo A Preti A Sæbye D Parnas J. Anomalies of imagination, self-disorders, and schizophrenia spectrum psychopathology: a network analysis. Front Psychiatry. (2022) 12:808009. 10.3389/fpsyt.2021.808009 35111092 PMC8801416

[2] Nordgaard J Henriksen MG Jansson L Handest P Møller P Rasmussen AR. Disordered selfhood in schizophrenia and the examination of anomalous self-experience: accumulated evidence and experience. Psychopathol. (2021) 54:275–81. 10.1159/000517672 34384082 PMC8686724

[3] Sass L Borda JP Madeira L Pienkos E Nelson B. Varieties of self disorder: a bio-pheno-social model of schizophrenia. Schizophr Bull. (2018) 44:720–7. 10.1093/schbul/sby001 29529266 PMC6007751

[4] Sass LA. Self-disturbance and schizophrenia: Structure, specificity, pathogenesis (Current issues, new directions). Schizophr Res. (2014) 152:5–11. 10.1016/j.schres.2013.05.017 23773296

[5] Parnas J Raballo A Handest P Jansson L Vollmer-Larsen A Saebye D. Self-experience in the early phases of schizophrenia: 5-year follow-up of the Copenhagen Prodromal Study. World Psychiatry. (2011) 10:200–4. 10.1002/j.2051-5545.2011.tb00057.x 21991279 PMC3188774

[6] IrarrÃ¡zaval L. The lived body in schizophrenia: transition from basic self-disorders to full-blown psychosis. Front Psychiatry. (2015) 6:9. 10.3389/fpsyt.2015.00009 25691874 PMC4315119

[7] Shergill SS Samson G Bays PM Frith CD Wolpert DM. Evidence for sensory prediction deficits in schizophrenia. Am J Psychiatry. (2005) 162:2384–6. 10.1176/appi.ajp.162.12.2384 16330607

[8] Stanghellini G. Embodiment and schizophrenia. World Psychiatry. (2009) 8:56–9. 10.1002/j.2051-5545.2009.tb00212.x 19293962 PMC2652898

[9] Zatti A Zarbo C. Embodied and exbodied mind in clinical psychology. A proposal for a psycho-social interpretation of mental disorders. Front Psychol. (2015) 6:236. 10.3389/fpsyg.2015.00236 25784894 PMC4347298

[10] Lysaker PH Vohs J Minor KS Irarrázaval L Leonhardt B Hamm JA. Metacognitive deficits in schizophrenia: presence and associations with psychosocial outcomes. J Nerv Ment Dis. (2015) 203:530–6. 10.1097/NMD.0000000000000323 26121151

[11] Fuchs T Schlimme JE. Embodiment and psychopathology: a phenomenological perspective. Curr Opin Psychiatry. (2009) 22:570–5. 10.1097/YCO.0b013e3283318e5c 19730373

[12] Pawełczyk A Kotlicka-Antczak M Łojek E Ruszpel A Pawełczyk T. Schizophrenia patients have higher-order language and extralinguistic impairments. Schizophr Res. (2018) 192:274–80. 10.1016/j.schres.2017.04.030 28438437

[13] de Boer JN Brederoo SG Voppel AE Sommer IEC. Anomalies in language as a biomarker for schizophrenia. Curr Opin Psychiatry. (2020) 33:212–8. 10.1097/YCO.0000000000000595 32049766

[14] Abbassi E Blanchette I Ansaldo AI Ghassemzadeh H Joanette Y. Emotional words can be embodied or disembodied: the role of superficial vs. deep types of processing. Front Psychol. (2015) 6:975. 10.3389/fpsyg.2015.00975 26217288 PMC4496550

[15] Graham SA Fisher SE. Understanding Language from a Genomic Perspective. Annu Rev Genet. (2015) 49:131–60. 10.1146/annurev-genet-120213-092236 26442845

[16] Wang H Pan Y. A brief review on embodied language comprehension. IJARP. (2015) 2:82–94. 10.46886/IJARP/v2-i1/2095

[17] Zwaan RA. The immersed experiencer: toward an embodied theory of language comprehension. In: The Psychology of Learning and Motivation: Advances in Research and Theory, Vol 44. New York, NY: Elsevier Science (2004). p. 35–62. 10.1016/S0079-7421(03)44002-4

[18] Zwaan RA Taylor LJ. Seeing, acting, understanding: Motor resonance in language comprehension. J Exp Psychol Gen. (2006) 135:1–11. 10.1037/0096-3445.135.1.1 16478313

[19] Vaskinn A Sundet K Østefjells T Nymo K Melle I Ueland T. Reading emotions from body movement: a generalized impairment in schizophrenia. Front Psychol. (2016) 6:2058. 10.3389/fpsyg.2015.02058 26834672 PMC4712298

[20] McCarthy-Jones S Krueger J Larøi F Broome M Fernyhough C. Stop, look, listen: the need for philosophical phenomenological perspectives on auditory verbal hallucinations. Front Hum Neurosci. (2013) 7:127. 10.3389/fnhum.2013.00127 23576974 PMC3620561

[21] Coulon N Godin O Bulzacka E Dubertret C Mallet J Fond G. Early and very early-onset schizophrenia compared with adult-onset schizophrenia: French FACE-SZ database. Brain Behav. (2020) 10:e01495. 10.1002/brb3.1495 31908151 PMC7010576

[22] First MB Williams JB Spitzer RL Gibbon M. Structured Clinical Interview for DSM-IV-TR Axis I Disorders, Clinical Trials Version (SCID-CT). New York: Biometrics Research, Newv York State Psychiatric Institute.(2007).

[23] Craddock N Asherson P Owen MJ Williams J McGuffin P Farmer AE. Concurrent validity of the opcrit diagnostic system: comparison of opcrit diagnoses with consensus best-estimate lifetime diagnoses. Br J Psychiatry. (1996) 169:58–63. 10.1192/bjp.169.1.58 8818369

[24] Khaleghzadeh-Ahangar H Talebi A Mohseni-Moghaddam P. Thyroid disorders and development of cognitive impairment: a review study. Neuroendocrinology. (2021) 28:521650. 10.1159/000521650 34963121

[25] Wang Y Lv J He J Wen G Wu X. Mechanism of psychoactive substance-induced cognitive disorders: does tau protein play a role? Front Biosci. (2022) 27:6. 10.31083/j.fbl2701006 35090311

[26] Liu X Yu Y Zhu S. Inflammatory markers in postoperative delirium (POD) and cognitive dysfunction (POCD): a meta-analysis of observational studies. PLoS ONE. (2018) 13:e0195659. 10.1371/journal.pone.0195659 29641605 PMC5895053

[27] Sheehan DV. The Mini-International Neuropsychiatric Interview (M.I.N.I.): the development and validation of a structured diagnostic psychiatric interview for DSM-IV and ICD-10. J Clin Psychiatry. (1998):59 (Suppl. 20):2212. 9881538

[28] Andreasen NC. Scale for the assessment of Thought, Language, and Communication (TLC). Schizophr Bulletin. (1986) 12:473–82. 10.1093/schbul/12.3.473 3764363

[29] Kay SR Fiszbein A Opler LA. The positive and negative syndrome scale (PANSS) for schizophrenia. Schizophr Bull. (1987) 13:261–76. 10.1093/schbul/13.2.261 3616518

[30] Perälä J Suvisaari J Saarni SI Kuoppasalmi K Isometsä E Pirkola S. Lifetime prevalence of psychotic and bipolar I disorders in a general population. Arch Gen Psychiatry. (2007) 64:19–28. 10.1001/archpsyc.64.1.19 17199051

[31] Corcoran CM Mittal VA Bearden CE Gur R Hitczenko K Bilgrami Z. Language as a biomarker for psychosis: a natural language processing approach. Schizophr Res. (2020) 226:158–66. 10.1016/j.schres.2020.04.032 32499162 PMC7704556

[32] de Boer JN van Hoogdalem M Mandl RCW Brummelman J Voppel AE Begemann MJH. Language in schizophrenia: relation with diagnosis, symptomatology and white matter tracts. NPJ Schizophr. (2020) 6:10. 10.1038/s41537-020-0099-3 32313047 PMC7171150

[33] Möller TJ Georgie YK Schillaci G Voss M Hafner VV Kaltwasser L. Computational models of the “active self” and its disturbances in schizophrenia. Conscious Cogn. (2021) 93:103155. 10.1016/j.concog.2021.103155 34130210

[34] Nordgaard J Gravesen-Jensen M Buch-Pedersen M Parnas J. Formal thought disorder and self-disorder: an empirical study. Front Psychiatry. (2021) 12:640921. 10.3389/fpsyt.2021.640921 33897496 PMC8060494

[35] Röder F Özdemir O Nguyen PDH Wermter S Eppe M. The embodied crossmodal self forms language and interaction: a computational cognitive review. Front Psychol. (2021) 12:716671. 10.3389/fpsyg.2021.716671 34484079 PMC8415221

[36] Jacob MS Ford JM Roach BJ Calhoun VD Mathalon DH. Aberrant activity in conceptual networks underlies N400 deficits and unusual thoughts in schizophrenia. Neuroimage Clin. (2019) 24:101960. 10.1016/j.nicl.2019.101960 31398555 PMC6699247

[37] Sharma A Sauer H Hill H Kaufmann C Bender S Weisbrod M. Abnormal N400 semantic priming effect may reflect psychopathological processes in schizophrenia: a twin study. Schizophr Res Treatment. (2017) 2017:7163198. 10.1155/2017/7163198 28932600 PMC5592423

[38] Berberian AA Moraes GV Gadelha A Brietzke E Fonseca AO Scarpato BS. Is semantic verbal fluency impairment explained by executive function deficits in schizophrenia? Braz J Psychiatry. (2016) 38:121–6. 10.1590/1516-4446-2015-1663 27096410 PMC7111369

[39] Lysaker PH. Schizophrenia, the self and psychotherapy: is there really anything new under the sun? J Clin Psychol. (2021) 77:1865–70. 10.1002/jclp.23234 34460959

