# Supplementary material for: Disembodied Language in Early-Onset Schizophrenia
Source: Front Psychiatry. 2022 Jul 5;13:888844. doi: 10.3389/fpsyt.2022.888844 (PMC9294146; doi:10.3389/fpsyt.2022.888844)
Supplement: Supplementary file 1 [file Table_1.DOCX]

Supplementary Material

# The procedure of Zabór Verbal Task (ZVT) for embodied language examination.

1. The patient is asked to stay in front of the board ( the board was set in the central part of the visual field, to resemble the external space), see part A of the figure below.
2. The patient is asked to read carefully all of the seven words from each set separately (the subsequent word group is presented after placing the previous one, words from the particular set are presented randomly). After reading the patient is asked to place each word on board “as it should be in her/his opinion”.

The exact command is: *Please read the word carefully and place one word in one square on the board as it should be placed following your opinion.*

1. The researcher notes word placing in a paper sheet with marked squares, similarly to the board.
2. Patient can change the word location during each set of words, the examination is not time-limited.
3. Words are put on the board using adhesive tape or a pin.

The word sets for each of three probes.

Set 1: Sky, bird, chair, cake, shoulders, grass, carpet

Set 2: Helicopter, sun, table, rice, stomach, floor, ground

Set 3: Cloud, plane, pasta, wardrobe, hand, road, root

Proper word location is presented in part B of the figure, an example of improper location in part C.

# Supplementary Figure

# Supplementary Figure 1. The procedure of Zabór Verbal Task (ZVT). A: location of the patient and the board during examination, with examples of proper word locations on the board (B) and improper word location (C).


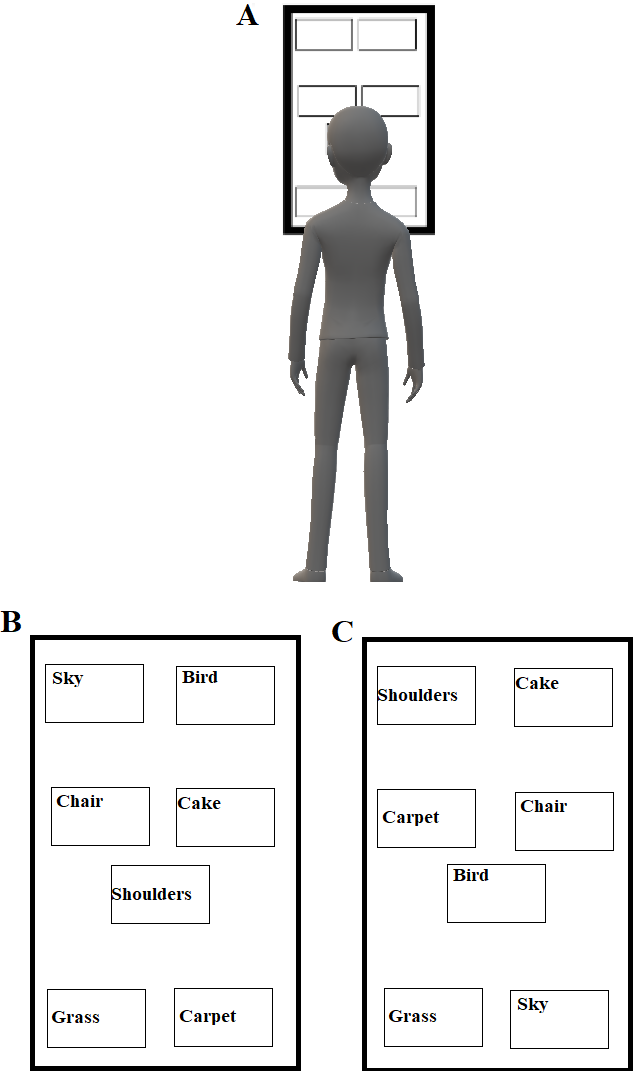


# Factor analysis

Factor analysis with Varimax rotation was performed: Table 1 (Tab.1).

Factor analysis revealed two factors. The threshold for inclusion of an item in one factor was 0.56. This threshold allowed to group each item exclusively in one dimension, leaving two unassigned items. Variance explained for Factor 1 was 5.66%, for Factor 2 - 5.38%

**Supplementary Table 1 (Tab.1)**

|  | \| **Factor 1** \| \| --- \| | \| **Factor 2** \| \| --- \| |
| --- | --- | --- | --- | --- |
| \| Shoulders \| \| --- \| | 0.815800 | 0.156306 |
| \| Pasta \| \| --- \| | 0.811805 | 0.010105 |
| \| Cloud \| \| --- \| | 0.730164 | 0.249162 |
| \| Rice \| \| --- \| | 0.687648 | 0.191161 |
| \| Carpet \| \| --- \| | 0.685430 | 0.242850 |
| \| Sun \| \| --- \| | 0.613932 | 0.518759 |
| \| Hand \| \| --- \| | 0.572854 | 0.024288 |
| \| Sky \| \| --- \| | 0.554272 | 0.411986 |
| \| Cake \| \| --- \| | 0.557094 | 0.358668 |
| \| Rad \| \| --- \| | 0.551596 | 0.462696 |
| \| Plane \| \| --- \| | 0.073218 | 0.786894 |
| \| Ground \| \| --- \| | 0.491495 | 0.767304 |
| \| Bird \| \| --- \| | 0.179564 | 0.744367 |
| \| Grass \| \| --- \| | 0.210020 | 0.646494 |
| \| Helicopter \| \| --- \| | 0.464358 | 0.644869 |
| \| Stomach \| \| --- \| | 0.308319 | 0.623349 |
| \| Chair \| \| --- \| | -0.029202 | 0.605458 |
| \| Wardrobe \| \| --- \| | 0.116493 | 0.587127 |
| \| Root \| \| --- \| | 0.469088 | 0.583856 |
| \| Table \| \| --- \| | 0.414788 | 0.451215 |
| \| Floor \| \| --- \| | 0.435653 | 0.418601 |
| \| **Variance explained (%)** \| \| --- \| | **5.657554** | **5.379719** |
